# Simultaneous Detection of *Sarcocystis hominis*, *S. heydorni*, and *S. sigmoideus* in Human Intestinal Sarcocystosis, France, 2021–2024

**DOI:** 10.3201/eid3103.241640

**Published:** 2025-03

**Authors:** Maxime Moniot, Patricia Combes, Damien Costa, Nicolas Argy, Marie-Fleur Durieux, Thomas Nicol, Céline Nourrisson, Philippe Poirier

**Affiliations:** CHU Clermont-Ferrand Service de Parasitologie-Mycologie, Clermont-Ferrand, France (M. Moniot); CHU Clermont-Ferrand Centre National de Référence des cryptosporidioses, microsporidies et autres protozooses digestives, Clermont-Ferrand (M. Moniot, P. Combes, D. Costa, C. Nourrisson, P. Poirier); Université Rouen Normandie, Rouen, France (D. Costa); CHU Rouen Centre National de Référence Cryptosporidioses, Microsporidies et autres protozooses digestives (Centre coordonnateur), Rouen (D. Costa); Hopital Bichat-Claude Bernard, Paris, France (N. Argy); MERIT UMR 216, IRD, Faculté de pharmacie de Paris, Université Paris Cité, Paris (N. Argy); Centre de biologie et de recherche en santé, Hôpital Universitaire Dupuytren, Limoges, France (M. Durieux); CHU Angers, Angers, France (T. Nicol); Microbes, Intestin, Inflammation et Susceptibilité de l’Hôte, UMR Inserm/Université Clermont Auvergne U1071, USC INRAE 1382, Clermont-Ferrand (C. Nourrisson, P. Poirier)

**Keywords:** parasites, enteric infections, zoonoses, intestinal parasite, Sarcocystis sigmoideus, Sarcocystis sp., molecular diagnosis, sarcocystosis, Sarcocystis hominis, Sarcocystis heydorni, intestinal sarcocystosis, France

## Abstract

To elucidate the epidemiology of *Sarcocystis* spp. parasites in human intestinal infections, we used high-throughput sequencing to investigate human intestinal sarcocystosis cases identified by microscopy in France during 2021–2024. Our results indicate that humans are a definitive host of *S. sigmoideus* parasites and that occurrence of multiple species in 1 patient is common.

The coccidian parasite *Sarcocystis* is one of the most frequently identified protozoa of warm-blooded and poikilothermic animals worldwide, causing an intestinal infection in the definitive host or an extraintestinal infection in the intermediate host (*1*). Human intestinal sarcocystosis (i.e., humans as the definitive host) is rarely reported (*2**–**6*); only 3 of nearly 200 described *Sarcocystis* species have been identified as responsible for human intestinal infections (*1*). Infection is acquired by ingesting raw or undercooked meat containing cysts of the parasite, such as pork for *S. suihominis* or beef for *S. hominis* and *S. heydorni* (*1*,*7*). One human case involving *S. cruzi* infection was also reported, but the presence of this species, for which canids are the definitive host, has yet to be confirmed in humans (*2*,*8*).

Genetic characterization of *Sarcocystis* spp. is commonly based on the mitochondrial cytochrome c oxidase subunit I (COI) gene sequence. Recently, Rubiola et al. described a new species that infects bovine muscle, named *S. sigmoideus* (*9*). Retrospective analyses of genomic data available in GenBank revealed that this species previously had been detected in 2 other carcasses in Italy and 6 in Belgium (*10**–**12*). The definitive host for this new species was still unknown (*9*). Here, we report the presence of *S. sigmoideus* sporocysts in feces from several human patients in France. We also report cases of *S. heydorni* infections and highlight a high frequency of patients infected with multiple species simultaneously. 

According to the French Ministry of Health, data for these patients were collected as part of routine surveillance and epidemiologic investigations by the National Reference Center for Cryptosporidiosis, Microsporidia and Other Digestive Protozoa (Public Health Code Article L 1413-3, https://www.santepubliquefrance.fr/a-propos/nos-principes-fondateurs/centres-nationaux-de-reference-pour-la-lutte-contre-les-maladies-transmissibles-cnr). Therefore, this study is exempt from institutional review board review.

## The Study

**Figure 1:**
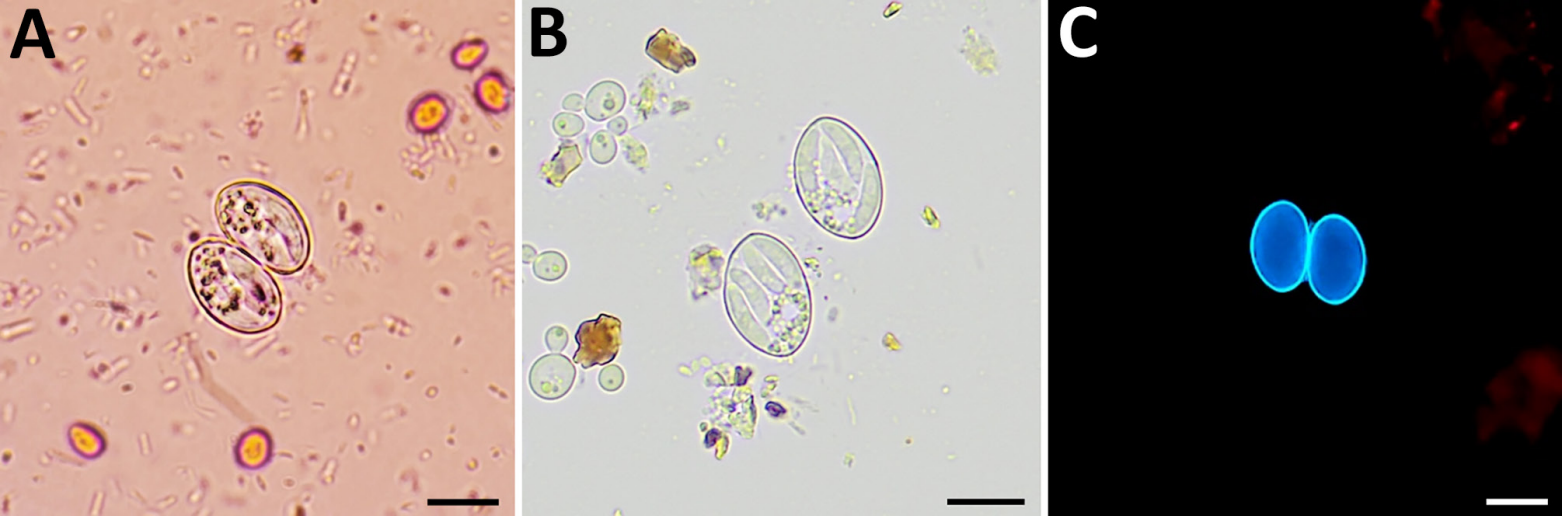
Oocysts of *Sarcocystis* spp. from patients with human intestinal sarcocystosis, France, 2021–2024. A) Concentrated stool smear stained using the merthiolate-iodine-formaldehyde method. Sporulated oocysts are colorless and contain 2 elongated sporocysts. The oocyst wall is thin and often invisible in wet mount. B) Wet mount. Each sporocyst contains 4 banana-shaped sporozoites and a granular sporocyst residuum, which may be compact or dispersed. The 4 sporozoites are rarely seen in a single plane of focus. C) Fresh homogenized stool smear under fluorescent microscopy. Individual sporocysts are autofluorescents and will appear blue with an excitation filter of 330–365 nm. Scale bars indicate 10 µm.

The patients included in this study underwent testing for intestinal parasites during October 2021–July 2024 because of gastrointestinal disorders or for systematic screening. Testing was performed in medical analysis laboratories by microscopic examination of fresh homogenized stool samples highlighting *Sarcocystis* spp. oocysts, sporocysts, or both (Figure 1). Oocysts/sporocysts were observed in 19 patients (Table), 8 women and 11 men, ranging in age from 19 to 94 years, all living in France. Of the 19 patients, 17 had reported acute, chronic, or occasional diarrhea lasting up to several months; the remaining 2 patients (case identification nos. S01-05 and S01-14) had infection diagnosed during systematic screening. No apparent cause other than *Sarcocystis* infection has been reported to explain the gastrointestinal disorders, except in 2 patients, 1 with concomitant *Salmonella* infection (case identification no. S01-03) and 1 with concomitant *Taenia saginata* infection (case identification no. S01-19). Some other symptoms were occasionally observed, such as abdominal pain, constipation, weight loss, nausea, ileitis, eosinophilia, or blood in stool (Table).

**Table:** Clinical manifestations and molecular findings for 19 cases of human intestinal sarcocystosis, France, 2021–2024*

Case ID	Age, y/sex	Signs/symptoms	Sample date	HTS		*Sarcocystis* species	GenBank accession no. of reference sequence
				Contigs	No. (%) reads		
S01-01	71/F	Acute diarrhea	2021 Oct 25	Contig 1	4,405 (83.2)	*S. sigmoideus*	OR543013
				Contig 2	730 (13.8)	*S. hominis*	OR543019
S01-02	94/F	Chronic diarrhea, alternating constipation	2022 Jan 22	Contig 1	2,470 (46.1)	*S. sigmoideus*	OR543013
				Contig 2	1,582 (29.5)	*S. hominis*	OR543021
				Contig 3	342 (6.4)	*S. hominis*	MK497842
S01-03	33/F	Acute diarrhea, abdominal pain, ileitis, *Salmonella* infection	2022 Aug 21	Contig 1	5,768 (64.7)	*S. hominis*	OR543021
				Contig 2	2,993 (33.6)	*S. hominis*	MK497842
S01-04	48/M	Abdominal pain, anal itching	2023 Feb 23	Contig 1	3,065 (56.1)	*S. hominis*	OR543021
				Contig 2	2,399 (43.9)	*S. hominis*	MK497842
S01-05	35/F	None†	2023 Mar 16	Contig 1	2,674 (73.4)	*S. hominis*	MK497842
				Contig 2	914 (25.1)	*S. heydorni*	KX057995
S01-06	73/M	Chronic diarrhea, abdominal pain, weight loss, eosinophilia	2023 May 9	Contig 1	4,767 (95.2)	*S. sigmoideus*	OR543013
				Contig 2	99 (2.0)	*S. hominis*	OR543021
S01-08	24/F	Acute diarrhea, nausea	2023 Jun 19	Contig 1	3,213 (68.5)	*S. hominis*	OR543019
				Contig 2	750 (16.0)	*S. heydorni*	KX057995
				Contig 3	673 (8.0)	*S. hominis*	MK497842
S01-09	76/F	Chronic diarrhea, abdominal pain	2023 Aug 30	Contig 1	2,787 (50.6)	*S. hominis*	OR543019
				Contig 2	1,881 (34.2)	*S. hominis*	MK497842
				Contig 3	603 (11.0)	*S. sigmoideus*	OR543013
S01-10	22/F	Chronic diarrhea, asthenia, multiple food intolerances, fibroscopic gastritis and rectitis	2023 Nov 14	Contig 1	5,049 (100)	*S. hominis*	OR543021
S01-11	19/M	Occasional diarrhea, sometimes blood in feces	2023 Nov 18	Contig 1	1,653 (39.5)	*S. sigmoideus*	OR543013
				Contig 2	1,049 (25.1)	*S. hominis*	OR543021
				Contig 3	927 (22.2)	*S. heydorni*	KX057995
S01-12	68/F	Chronic diarrhea, abdominal pain	2023 Dec 11	Contig 1	2,410 (48.8)	*S. hominis*	OR543021
				Contig 2	961 (19.4)	*S. hominis*	MK497842
				Contig 3	869 (17.6)	*S. sigmoideus*	OR543013
S01-13	26/M	Abdominal pain	2023 Dec 18	Contig 1	5,072 (99.9)	*S. hominis*	OR543021
S01-14	57/M	None‡	2024 Jan 9	Contig 1	4,246 (97.4)	*S. hominis*	OR543019
S01-15	79/M	Chronic diarrhea, abdominal pain, CDP 10 y prior	2024 Apr 3	Contig 1	1,428 (36.1)	*S. hominis*	OR543021
				Contig 2	767 (19.4)	*S. hominis*	MK497842
				Contig 3	696 (17.6)	*S. heydorni*	KX057995
				Contig 4	662 (16.7)	*S. sigmoideus*	OR543013
S01-16	62/M	Chronic diarrhea, abdominal pain	2024 Jun 12	Contig 1	1,171 (28.6)	*S. hominis*	MK497842
				Contig 2	1,153 (28.2)	*S. sigmoideus*	OR543013
				Contig 3	641 (15.7)	*S. heydorni*	KX057995
				Contig 4	590 (16.9)	*S. hominis*	OR543021
S01-18	73/M	Acute diarrhea, weight loss	2023 Jan 10	Contig 1	4,103 (100)	*S. hominis*	MK497842
S01-19	52/M	Chronic diarrhea, abdominal pain, weight loss, ulcerative colitis, *Taenia saginata* infection	2023 Sep 29	Contig 1	7,302 (100)	*S. hominis*	OR543019
S01-27	63/M	Abdominal pain, nausea, eosinophilia	2024 Jul 10	Contig 1	4,696 (99.9)	*S. hominis*	OR543021
S01-28	48/M	Chronic diarrhea, rectal cancer diagnosis	2024 Jul 22	Contig 1	3,976 (57.2)	*S. sigmoideus*	OR543013
				Contig 2	2,578 (37.1)	*S. hominis*	OR543021

*CDP, cephalic duodenopancreatectomy; HTS, high-throughput sequencing; ID, identification. †Preemployment medical screening of food industry worker. ‡Systematic screening in kidney transplantation.

**Figure 2:**
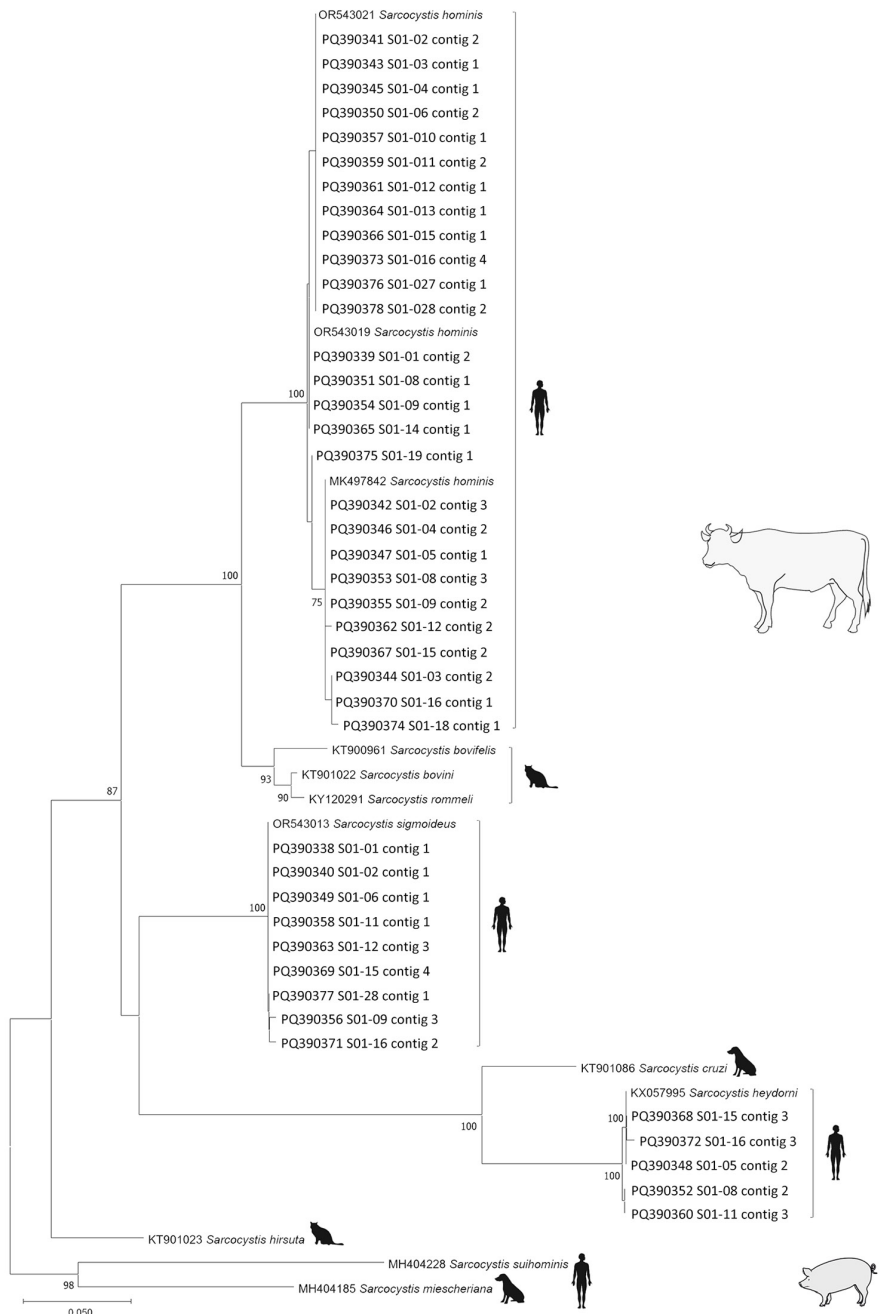
Phylogenetic tree for human *Sarcocystis* spp. from human intestinal sarcocystosis, France, 2021–2024. Tree is based on 54 partial mitochondrial cytochrome c oxidase subunit I gene sequences from patients compared with reference sequences from GenBank. The 41 sequences of 332 bp generated in this study are identified by patient numbers (i.e., S01-02); GenBank accession numbers are provided for reference sequences. This analysis included the 10 taxa described in pigs and cattle (intermediate hosts, blank illustrations) with the corresponding definitive host (humans, felids, or canids, black illustrations). The tree was inferred by using the neighbor-joining method and rooted on the species whose pigs serve as the intermediate hosts, *S. miescheriana* and *S. suihominis*. Evolutionary distances were computed using the Tamura-Nei method. Branch consensus support is expressed as percentage from 1,000 bootstraps and is reported next to the branches; branch support values <75% were not included. Scale bar indicates base substitutions per site.

A total of 23 stool samples from the 19 patients were prospectively sent to the French National Reference Center for Cryptosporidiosis, Microsporidia and Other Digestive Protozoa for further molecular analysis (Appendix). In brief, high-throughput sequencing (HTS) was performed on a 332-bp region of the mitochondrial cytochrome c oxidase subunit I gene. We constructed a phylogenetic tree on the basis of the partial gene sequence from the 41 characterized *Sarcocystis* spp. isolates and reference sequences from GenBank by using the neighbor-joining method (Figure 2). The different *Sarcocystis* spp. contigs clustered with the reference sequences with a maximum variation of 2 nt over the 332-bp sequence analyzed (Table). Most (11/19) patients were co-infected with multiple *Sarcocystis* species (*S. hominis* was most frequently detected); *S. sigmoideus* infection was detected in 9 patients and *S. heydorni* infection in 5 patients. 

## Conclusions

Among the *Sarcocystis* parasite species present in beef meat (i.e., cattle as intermediate host), *S. hominis* was the first species reported to infect humans as the definitive host (*8*). Then, *S. heydorni* was indirectly considered to infect humans after it was observed in calves fed with sporocysts from the feces of a human volunteer (*7*). Our results expand on that previous report of human *S. heydorni* infection by identifying 5 more human cases. Recently, *S. sigmoideus* was described as a novel species in bovine carcasses. Felids were hypothesized to be the definitive hosts for this species, whereas identical samples harboring both *S. sigmoideus* and *S. hominis* suggested a potential zoonotic role (*9*). Here, we confirm at least the second hypothesis by reporting that humans are a definitive host of *S. sigmoideus*. 

During the study period, we performed molecular analyses on microscopically positive fecal samples (i.e., detection of oocysts/sporocysts) from 19 patients and detected *S. sigmoideus* parasites in 9 of them. An association with *S. hominis* parasites was detected in 6 patients and with *S. hominis* plus *S. heydorni* parasites in 3 patients.

A limitation of our study is that we could not microscopically distinguish between sporocysts of different species because most patients were co-infected. We also had to consider that, after ingestion of infected meat, some transient *Sarcocystis* DNA resulted in HTS reads that were not associated with the sporocysts seen in the fecal samples. However, we excluded that possibility because repeated stools spaced over 3 to 24 days for 3 co-infected patients showed the same species in the same proportions (data not shown). Also, in the 19 cases analyzed, we never detected reads from *S. cruzi*, which is highly prevalent in beef meat but does not infect humans. To definitively confirm that hypothesis, future attempts should be made to perform single-cell sequencing on sporocysts/oocysts isolated from microscopy or by species-specific labeling with species-specific hybridization probes.

The prevalence of *S. sigmoideus* parasites in cattle has been reported to be low, but it is likely to be underestimated, as suggested by the number of infected patients (9 of 19) in our study (*9*). Further molecular studies in cattle and human stool are needed to better estimate the real prevalence of *S. sigmoideus* parasites.

We used HTS for molecular analysis of *Sarcocystis* spp. parasites in human stools and found that human intestinal sarcocystosis is mainly caused by multiple species simultaneously (11 of 19 patients were co-infected). That finding is in accordance with recent veterinary data that detected *Sarcocystis* spp. parasites in 64% of randomly sampled cattle carcasses and mixed infections of up to 3 species simultaneously (including *S. sigmoideus* and *S. hominis*) in 25% of intralesional samples and in 5.8% of extralesional samples from carcasses condemned because of the presence of bovine eosinophilic myositis (*9*,*10*).

*S. hominis* parasites are considered mildly pathogenic in humans, whereas *S. suihominis* infection is more virulent (*1*). However, data about *Sarcocystis* pathogenicity are scarce, outdated, and mostly derived from volunteers who ingested experimentally infected meat (*1*). The pathogenicity of *S. heydorni* and *S. sigmoideus* parasites is unknown, and further studies are required to address that issue. In conclusion, our data demonstrate that humans are a definitive host for *S. sigmoideus* parasites and that intestinal sarcocystosis frequently results from infection with multiple species.

Appendix Additional information on simultaneous detection of *Sarcocystis hominis*, *S. heydorni*, and *S. sigmoideus* in human intestinal sarcocystosis, France, 2021–2024.
