# Supplementary material for: Simultaneous Detection of Sarcocystis hominis, S. heydorni, and S. sigmoideus in Human Intestinal Sarcocystosis, France, 2021–2024
Source: Emerg Infect Dis. 2025 Mar;31(3):559–63. doi: 10.3201/eid3103.241640 (PMC11878308; doi:10.3201/eid3103.241640)
Supplement: Appendix — Additional information on simultaneous detection of Sarcocystis hominis, S. heydorni, and S. sigmoideus in human intestinal sarcocystosis, France, 2021–2024. [file 24-1640-Techapp-s1.pdf]

Article DOI: https://doi.org/10.3201/eid3103.241640

EID cannot ensure accessibility for supplementary materials supplied by authors. Readers who have difficulty accessing supplementary content should contact the authors for assistance.

Simultaneous Detection of *Sarcocystis hominis*, *S. heydorni*, and *S. sigmoideus* in Human Intestinal Sarcocystosis, France, 2021–2024

Appendix

Illumina sequencing of the *Sarcocystis* mitochondrial cytochrome c oxidase subunit I gene (COI mtDNA) in human stools.

DNA extraction and COI specific PCR

DNA was extracted from stools using the Quick-DNA Fecal/Soil Microbe Miniprep (ZymoResearch) following manufacturer recommendations. The COI mtDNA of *Sarcocystis* spp. was amplified by PCR with the newly designed primers COXF-NGSadapt (5′-TCGTCGGCAGCGTCAGATGTGTATAAGAGACAGggtatctttagygttgttggtactc-3′) and COXR-NGSadapt (5′-GTCTCGTGGGCTCGGAGATGTGTATAAGAGACAGcagtcvacggcctccgtatt-3′) containing the Illumina adapter sequences (capital letters) as well as the COI gene specific sequence (underlined letters).

Based on the alignment of NCBI reference sequences, the COI primer sequences were designed *in silico* to amplify at least the 9 following species of *Sarcocytis: S. bovini* KT901021, *S. rommeli* KY120292, *S. bovifelis* KT900992, *S. hirsuta* KT901077, S. *cruzi* KT901095, *S. heydorni* KX057995, *S. hominis* MK497840, *S. suihominis* MH404228, *S. sigmoideus* OR543025 (see alignment below). To assess reproducibility of our results, each sample was analyzed in two independent PCR and accuracy was assessed by the presence of one negative control for each set of 16 sample reactions. PCR was performed with the high fidelity Taq polymerase Platinum SuperFi II MasterMix (Invitrogen) under the following conditions: 5 µL of DNA, 1X Super Fi II master Mix, 0.5 µM of primers in a final volume of 25 µL. PCR cycling

was as follows: 1 min at 98°C, 40 cycles of 20 sec at 98°C, 20 sec at 56°C, 30 sec at 72°C and a final elongation of 5 min at 72°C.

HTS library preparation and sequencing

Amplifications were purified with the AMPure XP Beads (Beckman Coulter) following manufacturer recommendations and unique dual indexes (IDT for Illumina PCR Index Sets 1–4, 384 IDX; reference: 20043137) were added by using PCR on each purified product. Briefly, the PCR mix contained 7.5 µL of purified PCR product, 1X Super Fi II master Mix, 7.5 µL of indexes in a final volume of 40 µL and PCR program was: 3 min at 98°C, 8 cycles of 20 sec at 98°C, 30 sec at 60°C, 1 min at 72°C and a final elongation of 3 min at 72°C.

Indexed PCR products were then purified again with the AMPure XP Beads following manufacturer recommendations and 1µL was quantified on a Qubit 4 Fluorimeter with the DNA High Sensitivity kit (Qubit dsDNA HS assay, Invitrogen). Purified indexed PCR products were then pooled and normalized according to their concentration by mixing 150 ng of each PCR product and 20 µL of each negative control. The COI gene PCR fragments from patients were pooled with other similar PCR products from other projects to fill in a Miseq Nano kit v2 (MS-103–1003, Illumina), so up to 56 PCR products were pooled together. The pool was then quantified and diluted to 4 nM, denatured with 0.2 N NaOH and diluted to 10 pM in HT1 buffer following Illumina's MiSeq recommendations. Because of the low genomic diversity of the library, a high proportion of Phix sequence had to be added before loading on the sequencing cartridge. The MiSeq Reagent Nano Kit was therefore loaded with 250 µL of 8 pM library, 50 µL of denatured 20 pM Phix and 300 µL of HT1 and set for paired end reads of 249 bases. Under these conditions, we obtain 500 to 750 clusters/mm^2 with 25 to 30% of aligned Phix sequence and 5000 to 10000 merged reads per sample.

Sequence analysis

Sequences were then analyzed using Geneious Prime software (V2023.2.1). Reads were first paired, trimmed with BBDuk for adapters, low quality reads (<Q30) and discard of short reads (<200 bp). Reads were then merged and chimeric reads removed using Usearch. To preserve only the region between primers, reads were then mapped against a reference sequence (*i.e.* MK497840 *Sarcocystis hominis*) and cut to keep the 332 bp fragment. Geneious *de novo* assembler was then used to create contigs containing maximum 1% of mismatches. The

consensus sequences of each contig were generated using the majority base for each position. Co-infections with different species were considered if the percentage of sequences was superior to 2% of the number of reads used to create contigs and if the results were consistent between the two independent experiments. The negative controls did not show any reads similar to the COI mtDNA sequence.

Phylogenetic analyses

Evolutionary analyses were conducted in MEGA X. The tree was inferred using the neighbor-joining method and rooted on *Sarcocystis miescheriana* and *Sarcocystis suihominis*. The evolutionary distances were computed using the Tamura-Nei method. The number of bootstraps replication was 1000. This analysis involved 54 nt sequences.

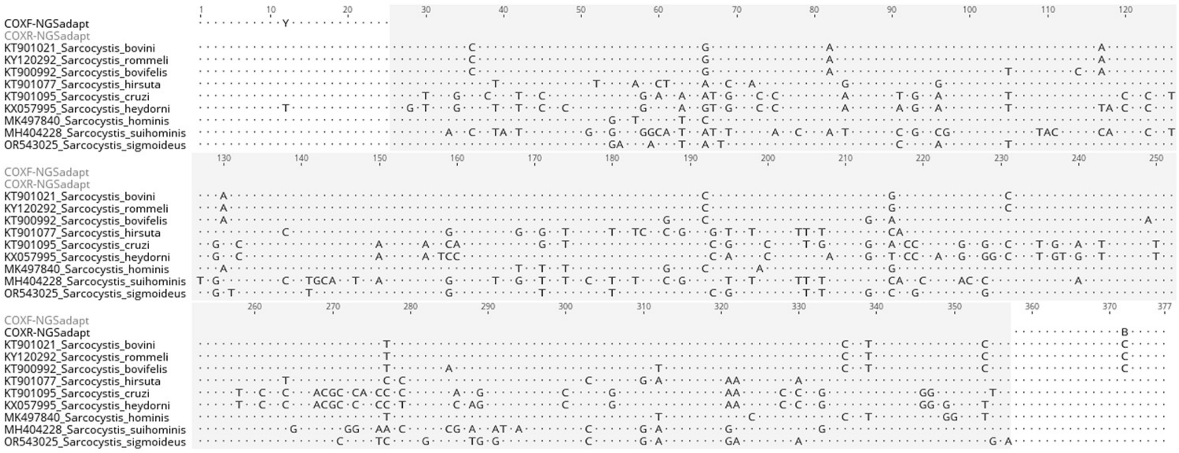

Appendix Figure. Pairwise alignment of COI mtDNA sequences from species used to design the primers. The 332 bp length region used for sequence analysis is highlighted in gray.
